# Cancer Specificity of Promoters of the Genes Involved in Cell Proliferation Control

**Published:** 2013

**Authors:** K. N. Kashkin, I. P. Chernov, E. A. Stukacheva, E. P. Kopantzev, G. S. Monastyrskaya, N. Ya. Uspenskaya, E. D. Sverdlov

**Affiliations:** Shemyakin-Ovchinnikov Institute of Bioorganic Chemistry , Russian Academy of Sciences, Miklukho-Maklaya St., 16/10, Moscow, Russia, 117997

**Keywords:** promoter, cloning, cancer-specific, cancer gene therapy

## Abstract

Core promoters with adjacent regions of the human genes CDC6, POLD1, CKS1B, MCM2, and PLK1 were cloned into a pGL3 vector in front of the Photinus pyrails gene Luc in order to study the tumor specificity of the promoters. The cloned promoters were compared in their ability to direct luciferase expression in different human cancer cells and in normal fibroblasts. The cancer-specific promoter BIRC5 and non-specific CMV immediately early gene promoter were used for comparison. All cloned promoters were shown to be substantially more active in cancer cells than in fibroblasts, while the PLK1 promoter was the most cancer-specific and promising one. The specificity of the promoters to cancer cells descended in the series PLK1, CKS1B, POLD1, MCM2, and CDC6. The bidirectional activity of the cloned CKS1B promoter was demonstrated. It apparently directs the expression of the SHC1 gene, which is located in a “head-to-head” position to the CKS1B gene in the human genome. This feature should be taken into account in future use of the CKS1B promoter. The cloned promoters may be used in artificial genetic constructions for cancer gene therapy.

## INTRODUCTION

The design of genetically engineered vectors that express products that are toxic for tumor cells holds an important position among the topical directions in the development of antitumor agents. These vectors need to contain cancer-specific regulatory elements that can ensure both the expression of the therapeutical gene in the maximum possible number of tumors and the absence of expression in normal tissues. Today, the number of promoters known to have these properties is limited.

While searching for new cancer-specific promoters, we have put forward a hypothesis that many promoters participating in DNA replication may exhibit tumor specificity, since the disturbed regulation of cell division is considered to be the common property of all tumors. In order to verify this hypothesis, we cloned the promoters of several genes participating in DNA synthesis and cell division and assessed the ability of these promoters to direct the expression of the reporter gene in normal and tumor cells of different origins. Promoters of the *CDC6, POLD1, CKS1B, MCM2, *and *PLK1* genes were used for cloning.

The *CDC6 *gene product is the homologue of *Saccharomyces cerevisiae *CDC6, a protein essential for the initiation of DNA replication. CDC6 regulates the early stages of DNA replication and helps control the check-point determining the termination of DNA replication before mitosis begins. A disturbed regulation of *CDC6 *expression is associated with a high risk of cancer development [1, 2]. The *POLD1 *gene encodes the catalytic subunit of DNA polymerase δ, which participates in the replication and reparation of human genomic DNA. This subunit exhibits polymerase (synthesis of DNA) and exonuclease (in the 3’–>5’ direction) activities. Moreover, POLD1 participates in the completion of the Okazaki fragments initiated by the DNA polymerase α/primase complex. The frequency of the development of spontaneous tumors is higher in mice with a deficient DNA polymerase δ function [3]. The CKS1B protein is a component of CDC28 protein kinase required for embryogenesis and correct alternation of the phases of the somatic cell cycle [4]. CKS1B forms a complex with the CDC2 protein and regulates the transcription of the *CDC20 *gene. The interaction between CKS1В and the SKP2–cyclin E-p27KIP complex ensures ubiquitination and degradation of p27 which is the cell blocker in the G0/G1 phase in response to different signals and unfavorable factors and the regulator of cell mobility and apoptosis [5]. The *CKS1B *gene localizes head-to-head with the *SHC1 *gene and presumably uses the bidirectional promoter shared with this gene [6]. *SHC1 *gene products are known to regulate the transfer of mitogenic signals in the cell, to participate in p53-dependent apoptosis under oxidative stress, and to regulat the lifespan. The protein р66Shc plays an important role in carcinogenesis and tumor dissemination [7]. The *MCM2 *gene encodes one of the subunits of the MCM2-7 protein complex, which is required for the initiation of DNA replication, formation of the replicative fork, and recruitment of the other proteins that participate in DNA replication. By interacting with the other proteins of the initiation complex, MCM2 regulates its helicase activity [8]. The promoters of the *SHC1 *and* MCM2 *genes are not characterized yet in detail. PLK1 (polo-like kinase 1) – serine/threonine protein kinase 1 – has several crucial functions during the M-phase of the cell cycle, including centrosome maturation, mitotic spindle assembly, and regulation of mitotic exit and cytokinesis. The PLK1 protein is required for cell restoration after DNA damage and when it enters mitosis [9]. The listed properties of the six genes and their increased expression in a number of human tumors (GeneHub GEPIS, [10]) provide grounds for hoping that the selected promoters could exhibit both tumor specificity and versatility with respect to tumors and would be able to act as regulator elements within genetically engineered anti-tumor constructs.

## EXPERIMENTAL

Promoters were amplified from the human genomic DNA using Tersus and Encyclo DNA polymerases (Evrogen, Russia). Promoters were cloned in the given coordinates with respect to the transcription start site (TSS) using the primers listed in *Table*. All the primers were synthesized on an ABI 3900 synthesizer (Applied Biosystems). The amplified DNA fragments were cloned into the vector in pAL-TA (Evrogen, Russia) and re-cloned into the pGL3 Basic Vector (Promega, WI, USA) at the proper restriction sites in front of the *Photinus pyralis* luciferase gene. Plasmid clones containing promoters in the required orientation were selected by restriction analysis and DNA sequencing. The resulting clones with promoters of the *CDC6, CKS1B, *and *PLK1 *genes contained no nucleotide substitutions, while the clones with the promoters *POLD1 *and *MCM2 *contained one and two substitutions with respect to the nucleotide sequences, respectively, which are listed in NC BI GenBank. We used the plasmid clones containing the cloned promoters to transfect the following cell lines: A375 (malignant melanoma, ATCC ), A431 (epidermoid carcinoma of the skin, ATCC ), A549 (lung carcinoma, ATCC ), Calu1 (lung epidermoid carcinoma, EC ACC ), HepG2 (hepatocellular carcinoma, ATCC ), HT1080 (fibrosarcoma, ATCC ), Panc-1 (epithelioid pancreatic carcinoma, ATCC ), and normal fibroblasts IVL-7C. Fibroblasts IVL-7C were obtained from the morphologically normal tissue of the lung of a patient who had undergone surgical resection of his lung cancer at the Blokhin Cancer Center, Russian Academy of Medical Sciences, using the previously described procedure [11]. Co-transfection with the plasmid pRL-TK (Promega, WI, USA) expressing the *Rluc *gene was used as an internal control of the transfection. Parallel transfection of cells with the vectors pGL3 Basic Vector, pGL3 Promoter Vector (Promega, WI), and pGL3- CMV Pr/Enh containing the AseI/BglII fragment of the promoter of early cytomegalovirus genes from the plasmid pEGFP-N1 (Clontech Laboratories, Inc.) in front of the *Luc *gene was used to standardize the experimental results. In order to compare the tumor specificity of the promoters, the cells were transfected with the 1500-bplong pGL3-based plasmid containing the promoter of the surviving gene (*BIRC5) *[12]. The cells were transfected by means of Lipofectamine 2000 (Invitrogen, USA) in 24-well plates according to the manufacturer’s recommendations. Promoter activity was assessed from the chemiluminescence of cell extracts. The chemiluminescence was measured using a Dual Luciferase Reporter Assay System (Promega, USA) on a GEN ios Pro plate luminometer (Tecan, Switzerland). The luminescence values of *P. pyralis *luciferase were standardized for the luminescence of *Renilla reinformis *luciferase in each measurement, and a correction for the background activity of the luciferase for the plasmid pGL3 Basic Vector was introduced. The resulting values were averaged for two repeats in each experiment and for a series of three experiments. The data were standardized for the* P. pyralis *luciferase activity under the control of the SV40 promoter within the pGL3 Promoter Vector.

**Table:** Primers used for promoter amplification

Promoter	Primer (5’ – > 3’)
POLD1 (–1338; +66)*	GGTACCTGAATACAATCCAGCCCGGAG GGTACCCCTCTACTCACCCGCTTCAAAC
CDC6 (–1539; +238)	GCTAGCGATCATGGCACGGCACTCA GCTAGCTCAGACCTCCAGCGAGCTCA
CKS1B (–910; +106)	GGTACCGGTCCCACAAAGATAAAGCTCC GGTACCTATGATCGCTCGGTTTGCTAG
MCM2 (–1949; +57)	ATCCGAGGTGCATCCTTCAC AGCAGTACCACGATCCTCTCC
PLK1 (–2338; +35)	GCAAGACTCCATCTCAACAACA CAGACCTCGATCCGAGCAG

* Coordinates of promoter with respect to the transcription start site of the gene.

**Fig. 1:**
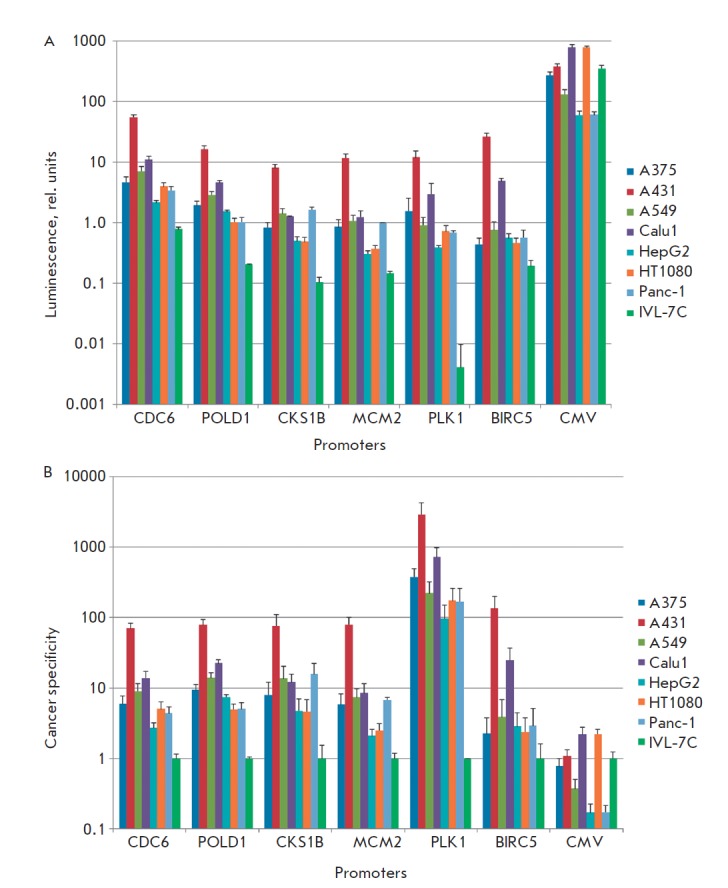
Activity and cancer specificity of cloned promoters. A – Chemiluminescence of lysates of cells transfected with plasmids with the corresponding promoters (logarithmic scale). The mean values and standard errors of the mean (SEM) are presented. B – Cancer specificity of promoters expressed as ratios between the chemiluminescence levels of the lysates of cancer cells and fibroblasts for each promoter. M – the median of the ratios, generalized index of cancer specificity of promoter

**Table:** 

Promoter	CDC6	POLD1	CKS1B	MCM2	PLK1	BIRC5	CMV
M	5.95	9.37	12.19	6.80	220.00	2.94	0.78

* Coordinates of promoter with respect to the transcription start site of the gene.

## RESULTS AND DISCUSSION

It was demonstrated in the transfection experiments that the cloned promoters exhibited activity in all the cell types under study. It should be mentioned that the activity of the cytomegalovirus promoter (CMV) within the construct pGL3-CMV Promotor/Enhancer Vector was 100- to 1,000-fold higher as compared to that of all the other promoters. The activities of promoters of the *POLD1, CDC6, CKS1B, PLK1 *and *MCM2 *genes in all the human tumor cell lines turned out to be higher than that of the SV40 promoter and were comparable to that of the *BIRC5 *gene promoter (*Fig. 1A*). Meanwhile, the promoters under study (except for the CMV promoter) in normal fibroblasts ensured a considerably lower level of luciferase activity as compared to the SV40 promoter. In order to assess the cancer specificity of each cloned promoter, we calculated the ratio between the chemiluminescence levels of the lysates of tumor cells and fibroblasts transfected with plasmids with the corresponding promoters, and the median value of these ratios as the generalized index of the cancer specificity of the promoter (*Fig. 1B*). The activity exhibited by the *BIRC5 *gene promoter, similar to that in the previous study [12], was higher in all the tumor cell lines as compared to that in normal fibroblasts. The activity of five cloned promoters in the tumor cells was also higher than that in normal fibroblasts (*p * < 0.01, Mann–Whitney U test). The CMV promoter exhibited no specificity: its activity in some tumor cells was higher as compared to that in fibroblasts, while in other tumor cells it was lower.

**Fig. 2:**
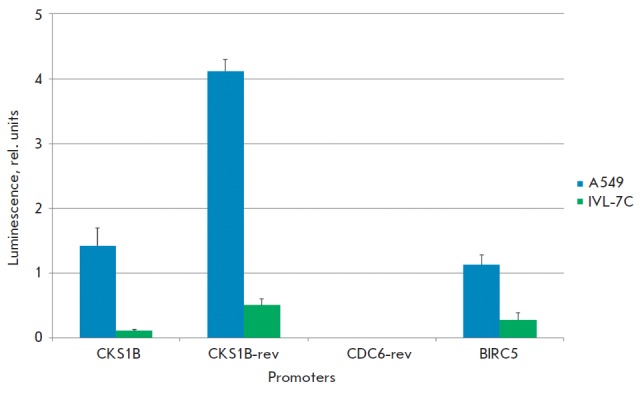
Chemiluminescence of the lysates of A549 and IVL-7C cells transfected with pGL3-based plasmids with promoters in direct and reverse (rev) orientations. The mean values and the standard errors of the mean (SEM) are presented. See text for explanations

As mentioned previously, the *CKS1B *and *SHC1* genes have a head-to-head orientation and presumably share one bidirectional promoter [6]. Hence, when cloning the *CKS1B *promoter, we additionally selected the pGL3 plasmid clone containing this promoter in reverse orientation. The coordinates of the cloned DNA fragment with respect to the TSS of the *SHC1 *gene were (–264; +751). In order to verify the promoter activity of the cloned fragment, we used this clone to transfect A549 cells and normal fibroblasts and measured the chemiluminescence of the cell lysates. The results are shown in *Fig. 2*. The results of determining the activity of the incorporated *CDC6 *promoter with the reverse orientation (*CDC6-rev*), which was identical to that of the control vector pGL3-BV, and the *BIRC5 *promoter in direct orientation are given here for the sake of comparison. It turned out that the activity of the cloned* SHC1 *promoter was higher than that of the *CKS1B* promoter both in the tumor cells and in normal fibroblasts. The activity of the *SHC1 *promoter in A549 adenocarcinoma cells was approximately eightfold higher than that in normal fibroblasts, which is lower than the activity of the *CKS1B *promoter but is comparable to the cancer specificity of the *BIRC5 *promoter (not shown). Since the range of tumors with an increased expression level of the *SHC1 *gene is smaller than that of the tumors with an increased expression level of the other genes that were used in our work (GeneHub GEPIS, [10]), we did not study the *SHC1 *promoter using other cells.

Thus, the promoters of five genes that regulate DNA replication and cell division, exhibiting tumor-specific expression, and significantly contribute to carcinogenesis had been cloned. When cells were transfected with plasmid vectors expressing the luciferase gene under the control of these promoters, the promoters exhibited a considerably higher activity in tumor cells of different origins as compared to their activity in normal fibroblasts. The activity and tumor specificity of the cloned promoters, except for the *PLK1 *promoter, was comparable to the indices for the *BIRC5 *promoter that had been studied previously. The specificity of the promoters slightly decreased for the series *CKS1B, POLD1, MCM2, CDC6*. The *PLK1 *promoter exhibited *considerably higher cancer specificity; the expressionlevels of the reporter gene controlled by the PLK1 andBIRC5 *promoters in the tumor cells were approximately *identical. This makes the PLK1 promoter superior toother promoters and provides grounds to regard it asthe most promising promoter for designing geneticallyengineered anti-tumor constructs.*

We have also demonstrated the bidirectional activity and high cancer specificity of the cloned *CKS1B/SHC1* promoter. One should take these features into account when designing genetically engineered vectors with this promoter, since its bidirectional activity may result in undesirable transcription of the vector sequences in a direction reverse to that of the therapeutical gene. On the other hand, this promoter can be used to simultaneously express two therapeutical genes in tumors or to design anti-tumor constructs that have binary effect with a more complex regulation. Further investigation into the *CKS1B/SHC1 *promoter and the genes whose expression it directs in various tissues and tumors is required.

It should be mentioned that only non-tumor control (normal lung fibroblasts) was used in this study. Taking into account the source of the cells (normal tissue obtained from a patient with lung cancer) and the fact that the properties of cells dividing in culture may differ from their properties *in vivo*, one needs to study the cloned promoters in *in vivo *models in order to draw unambiguous conclusions about the cancer specificity of the promoters.

The significant length of the promoters (1016–2373 bp) allows one to put forward a hypothesis that they contain key elements in transcription regulation, such as core promoters and proximal regulatory elements. However, it is entirely possible that there are additional remote regulatory elements, such as enhancers, silencers or repressors, which also participate in the regulation of the activity of these promoters. A comparison of the endogenous activity of the corresponding genes in various cell lines and tissues with the results obtained in our study will allow one to assess the relative contribution of the promoter and additional regulatory elements.

## Abbreviations

TSS: transcription start site
